# Structure alignment based on coding of local geometric measures

**DOI:** 10.1186/1471-2105-7-346

**Published:** 2006-07-14

**Authors:** Peter L Chang, Andrew W Rinne, T Gregory Dewey

**Affiliations:** 1 Keck Graduate Institute of Applied Life Sciences, Claremont, CA, USA

## Abstract

**Background:**

A structure alignment method based on a local geometric property is presented and its performance is tested in pairwise and multiple structure alignments. In this approach, the writhing number, a quantity originating from integral formulas of Vassiliev knot invariants, is used as a local geometric measure. This measure is used in a sliding window to calculate the local writhe down the length of the protein chain. By encoding the distribution of writhing numbers across all the structures in the protein databank (PDB), protein geometries are represented in a 20-letter alphabet. This encoding transforms the structure alignment problem into a sequence alignment problem and allows the well-established algorithms of sequence alignment to be employed. Such geometric alignments offer distinct advantages over structural alignments in Cartesian coordinates as it better handles structural subtleties associated with slight twists and bends that distort one structure relative to another.

**Results:**

The performance of programs for pairwise local alignment (TLOCAL) and multiple alignment (TCLUSTALW) are readily adapted from existing code for Smith-Waterman pairwise alignment and for multiple sequence alignment using CLUSTALW. The alignment algorithms employed a blocked scoring matrix (TBLOSUM) generated using the frequency of changes in the geometric alphabet of a block of protein structures. TLOCAL was tested on a set of 10 difficult proteins and found to give high quality alignments that compare favorably to those generated by existing pairwise alignment programs. A set of protein comparison involving hinged structures was also analyzed and TLOCAL was seen to compare favorably to other alignment methods. TCLUSTALW was tested on a family of protein kinases and reveal conserved regions similar to those previously identified by a hand alignment.

**Conclusion:**

These results show that the encoding of the writhing number as a geometric measure allow high quality structure alignments to be generated using standard algorithms of sequence alignment. This approach provides computationally efficient algorithms that allow fast database searching and multiple structure alignment. Because the geometric measure can employ different window sizes, the method allows the exploration of alignments on different, well-defined length scales.

## Background

As the number of protein structures continues to grow, structure comparison techniques have become an increasingly crucial bioinformatics tool. Because protein structures evolve more slowly than protein sequences, structure comparison can be used to assess distant evolutionary relationships and common functions for pairs that do not have high sequence similarity (cf. [1]). Structure alignment is also an important tool for protein classification and structural genomic initiatives. Despite the importance of structure comparison, a number of fundamental issues remain unresolved. One of the central problems is the mathematically difficult task of scoring and optimizing the structural alignment of three-dimensional objects. Most protein comparison algorithms treat proteins as rigid bodies and measure the quality of the superposition using distance-based measures such as RMSD (Root Mean Standard Deviation). Even after considerable effort [2-6], no algorithm has emerged as the method of choice for structure alignment [7].

Rigid body superpositions with distance metrics are less than optimal because subtle twists or bends in a protein structure can have a profound influence on the scoring of the alignment. These are often corrected by considering local alignments or introducing gap penalties. Recently, a number of new algorithms have been developed that allow for the flexible alignment of local fragments [8,9]. A particularly difficult comparison is presented by proteins with a hinge that separates similar domains. A slight alteration in the hinge can result in an alignment favoring the superposition of one domain over the other. Distance-based measures suffer from a fundamental limitation in that they do not obey the triangle inequality (cf. [10]). That is: similarity between proteins 1 and 2 and proteins 1 and 3 does not imply similarity between proteins 2 and 3. This issue is particularly prominent in proteins composed of distinct, separate domains. Because of the failings of distance-based metrics, a number of alternative, topological metrics have been proposed [10-12]. These approaches define a family of global geometric measures based on knot theory and use them to develop protein structure classification schemes. These measures use Gauss integrals to calculate the equivalent of the Vassiliev knot invariant for an open polygonal curve. Unlike the invariants of topology, these quantities are not fully invariant upon deformation and are represented by numerical values rather than integers.

The use of such measures is similar in spirit to earlier work on the differential geometry of proteins (see [13] for a review and [14] for a recent application). Differential geometric approaches define a geometric variable along 4 or 5 α-carbons length scales. Since the φ-ψ angles of the peptide bond is a local measure and protein contact is a global measure of structure, the differential geometric approach was deemed an attractive tool to probe intermediate length scales. The writhing number is an even more versatile quantity obtained from the theory of knots that can extend the length scale under consideration to include all length scales greater than 4 α-carbons. The attraction of this approach is that it provides a metric for virtually all length scales of the protein under a single Gauss integral metric.

In the present work, we extend the consideration of non-distance related metrics to develop algorithms for structure alignment. The writhing number is used as a local geometric measure that describes the curvature of the protein backbone formed from short connected segments of α-carbon atoms. Originally defined to describe the topology of closed circular DNA, the definition of the writhing number has recently been extended to consider open polygonal chains. Using a sliding window, the writhing number is calculated along successive regions of the chain. This calculation provides a local geometric profile of each protein. The regions considered in this work encompass 4, 5, 6 and 10 α-carbons. The values for the writhing number at each different length scale are separately encoded into a 20-letter alphabet by partitioning the histogram of all segment values obtained from RCSB Protein Data Bank (PDB) into bins and assigning each bin a letter in the alphabet. This procedure allows standard sequence alignment algorithms to be used to compare the geometric profiles. Using this approach, we have successfully "re-sequenced" all 52,087 proteins available in the PDB at the time of this work and have stored them into our own database for quick access. Using a block alignment approach identical to that used in calculating the BLOSUM substitution matrix, a scoring matrix for substitutions in the geometric alphabet was determined. Using this matrix (referred to as TBLOSUM) and our resequenced structure data bank, standard sequence alignment methods were used to perform structure alignments. To validate this approach, the performance of the local Smith-Waterman alignment (TLOCAL) and the CLUSTALW (referred to as TCLUSTALW) were used to perform high quality pairwise alignment and multiple structure alignment, respectively. This performance compares favorably with existing methods.

## Results

### Pairwise alignment of "difficult" structures

Using a database of sequences encoded from writhing numbers and a block scoring matrix (see Methods), several test proteins were selected to optimize the performance of TLOCAL and compare it to other methods. Alignments of ten "difficult" pairs of structure [15] were explored. A "difficult" pair is a structurally-similar pair of low sequence similarity which had proven difficult to align with the methods available at the time. We sought to optimize the gap penalties and sliding window size for the alignments. The TBLOSUM matrix was constructed with a sequence gap penalty designated (4,1) where 4 is the gap initiation penalty and 1 is the gap extension penalty. Varying the gap parameters with a sliding window of 5 showed that gap penalties of (2,2), (4,1) and (4,2) performed comparably and were very dependent on the specific example. In all subsequent work penalty parameters of (4,1) were used.

Table 1 shows the performance of the TLOCAL algorithm for different size windows for the ten "difficult" pairs [see Additional file 1]. This table shows the alignment scores as well as a distance metric, the Aligned Fragment Pairs Root Mean Squared Deviation (AFPRMSD). It is not straightforward to compare our geometric alignments with those obtained from distance-based methods because the geometric alignments do not provide a specific three-dimensional representation of the alignment. Rather the method creates pairs of similar local topology. To compare our method with sequence based methods we take aligned sequence pairs (AFP) and use standard methods to perform a local alignment (see Methods). As can be seen from the table, the performance of window sizes 4, 5 and 6 are comparable and are generally preferable to window size 10. There can be considerable difference in the number of pairs aligned with different window sizes. While the topological score generally agrees with the AFPRMSD, counterexamples to this are easily found.

In Table 2, the quality of the alignments as measured by AFPRMSD is compared for TLOCAL's (window size of 5), CE and FATCAT [see Additional file 2]. Direct comparison of the AFPRMSD is not possible because the number of aligned pairs changes from one method to another. While it is possible to adjust the aligned pairs in TLOCAL by changing the gap penalties, this approach would not necessarily assure an optimal scoring alignment. Recently, the dependence of RMSD on alignment length, N, has been demonstrated to scale as: *RMSD *∝ *N*^1/3 ^[16]. The radius of gyration of a protein, *R*_*g *_also scales as: *R*_*g *_∝ *L*^1/3 ^where L is the protein length [17]. From these observations a dimensionless quantity, the *reduced AFPRMSD *is defined as:

reducedAFPRMSD=AFPRMSDa0N1/3     (1)
 MathType@MTEF@5@5@+=feaafiart1ev1aaatCvAUfKttLearuWrP9MDH5MBPbIqV92AaeXatLxBI9gBaebbnrfifHhDYfgasaacH8akY=wiFfYdH8Gipec8Eeeu0xXdbba9frFj0=OqFfea0dXdd9vqai=hGuQ8kuc9pgc9s8qqaq=dirpe0xb9q8qiLsFr0=vr0=vr0dc8meaabaqaciaacaGaaeqabaqabeGadaaakeaacqWGYbGCcqWGLbqzcqWGKbazcqWG1bqDcqWGJbWycqWGLbqzcqWGKbazcqWGbbqqcqWGgbGrcqWGqbaucqWGsbGucqWGnbqtcqWGtbWucqWGebarcqGH9aqpdaWcaaqaaiabdgeabjabdAeagjabdcfaqjabdkfasjabd2eanjabdofatjabdseaebqaaiabdggaHnaaBaaaleaacqaIWaamaeqaaOGaemOta40aaWbaaSqabeaacqaIXaqmcqGGVaWlcqaIZaWmaaaaaOGaaCzcaiaaxMaadaqadiqaaiabigdaXaGaayjkaiaawMcaaaaa@513F@

and for simplicity we set *a*_0 _to 1 Å. This quantity is now used in Tables 1-3 to compare all alignments of different lengths. In all cases, the reduced AFPRMSD shows that TLOCAL outperforms FATCAT in all cases and CE in 6 out of 10 cases [see Additional file 1, 2, 3]. One should bear in mind that CE and FATCAT were not designed to optimize the score of an alignment calculated in this fashion.

### Pairwise alignment of hinged proteins

As an additional assessment of the performance of the local topological alignment algorithm, the performance on the alignment of structures with flexible or hinged regions was determined. The difficulty in aligning proteins with hinged regions motivated the development of new structural alignment programs; FATCAT [9] and FlexProt [8] that identified aligned AFPs and separately align these regions. The geometric alignment program is expected to perform well in these examples because the displacement caused by hinges do not affect the topology on either side of the hinge and should, therefore, allow for good alignment in these regions. Table 3 shows the performance of TLOCAL alignment for 16 different hinged structures that were examined previously [8,9] [see Additional file 3]. As can be seen, TLOCAL with window sizes of 4 outperforms FATCAT in all cases when considering reduced AFPRMSD. There are, however, instances when FATCAT performs better for the other TLOCAL window sizes.

### Multiple sequence alignment on a kinase superfamily

In addition to pairwise alignments performed by TLOCAL, the performance of the multiple structure alignment program (TCLUSTALW) was also examined. To evaluate the performance of TCLUSTALW, a family of protein kinases was aligned and the identified conserved regions were compared with those determined previously by a hand alignment. These 25 sequences include serine/threonine and tyrosine kinases provided by Scheeff and Bourne [18] that are representative of typical protein kinases (TPK). These kinases are found in different source species including human, pig, cow, rat, rabbit, baker's yeast, corn, and bacterial species. The hand alignment presented previously also included six structures known as atypical kinases. These structures were not considered here because they are not derived from protein kinases, but are believed to have evolved early in the evolutionary timescale to a convergent functional structure [18].

The comparison of hand alignments and those resulting from TCLUSTALW are shown side-by-side in Table 4 [see Additional file 4]. The alignment positions shown in red indicate those positions whose residues are highly conserved or exhibit extremely similar chemical-physical properties such as hydrophobicity or charge. Of the 24 alignment positions highly conserved as previously noted [18], 17 alignment positions were aligned correctly by TCLUSTALW for all residues at that position. The remaining 7 positions also had a strong consensus with only 1, 2, 3, or 6 deviations among the 25 aligned proteins. In addition to these 24 highly conserved alignment positions, Scheeff and Bourne [18] also note 23 alignment positions shown in gray whose residues are less conserved, but still exhibit similar chemical-physical properties. TCLUSTALW was able to align many of these residue positions as well.

## Discussion

In this work, a geometric profile of an individual protein is created by calculating the writhing number of consecutive segments (sliding window) along the protein chain. The profile is then encoded into a geometric alphabet by associating a range of numerical values with different letters of the alphabet. This alphabet is determined by observing the histogram of the frequency of writhing values in all segments of all the proteins observed in the PDB. This histogram is partitioned into bins and a letter from the geometric alphabet is associated with each bin. The numerical range of the bins is adjusted so that each bin contains the same number of segments. Thus, if a segment is chosen at random, it would have an equal chance at falling into any one bin. Consequently, each letter in the "geometric alphabet" has an equal chance of occurring in a protein structure. The motivation of partitioning the histogram in this fashion is to maximize the information content of the alphabet. Other ways of encoding the writhing number could conceivably be more effective. For instance, some geometric features may be more relevant or distinctive than others and it might be important to carefully delineate the values of the writhing numbers associated with these features. Such level of detail has not been investigated to date and lacking such information, the maximum information entropy approach is taken as a good first approach to encoding the local topological information in the protein profile.

A second important issue is the size of the alphabet used to encode the writhing number, a continuous variable. In principle, the smaller the bin range the greater the information content. In the limit of the bin size approaching the inherent error in the writhing number, more information will no longer be captured by decreasing the bin size. This error limit could be obtained by the propagation of the experimental error of the α-carbon atom positions used in the calculation of the writhing number. However, in mapping the structural alignment problem into a sequence alignment problem, not only is an accurate encoding required but also an accurate scoring system must be obtained. As the alphabet is expanded, more data is needed to accurately determine the values of the substitution matrix. Additionally, the programs calculating alignments will become increasingly computationally intense. There will be a trade off between increasing resolution of the bins of the histogram and the concomitant loss of scoring accuracy and increase of computation intensity. Again, these issues have not as of yet been explored in depth. Our strategy has been to adopt the twenty letter alphabet common to existing protein sequence alignment and to investigate the performance of the topological alphabet and scoring system under these familiar conditions. Keeping with these conditions, the gap penalties are treated as adjustable parameters and are generally in the range of values used for sequence alignment.

Given these conditions, the structure alignment matches local geometric propensities between different proteins and aligns the topological sequence to optimize the score from these propensities. As such, no Cartesian spatial associations can be directly assigned to these alignments. This topological association rather than a direct physical association is at the heart of the method and allows the alignment to avoid the difficulties with spatial alignment of rigid bodies as exemplified by the problem with hinged proteins. While the geometric alignment method does not allow for the familiar three-dimensional viewing employed in most existing structural alignment algorithms, this approach directly addresses the deeper issue of comparing similar structural regions that are offset by intervening differences. The problem of properly assigning alignments on either side of a hinge region is then approachable by this method.

Difficulties such as those presented by hinged proteins call to question the very nature of the structure alignment problem. Several authors have suggested that the alignment problem as commonly posed is not a well-defined problem and may not have an optimal solution (cf. [19]). Alignment methods seek to identify a biologically relevant correspondence between an amino acid residue in one protein with that in another. A variety of structural features from local orientation to global positioning may bear on this correspondence. The difficulty that is inherent in alignment methods is that they must in some sense be scale free, offering the most relevant correspondence across all length scales. This feature results in conflicting optimization criteria. The present method does not provide a solution to this general problem. Rather it provides a set of optimal solutions as defined by dynamic programming for a set of length scales as given by the sliding window size. Thus, the method offers an optimization solution for a well-defined length scale and should be interpreted in those terms.

To allow comparison with methods that use distance metrics as a measure of alignment quality, we employed the device of identifying AFPs from the topological alignment and using these segments as rigid bodies for a local structural alignment. The RMSD could then be calculated from the sum of all these local alignments. Using this measure, we observe that the Smith Waterman topological alignment, TLOCAL, compares favorably with CE and with FATCAT for both "difficult" protein pairs and for hinged proteins. This demonstrates the versatility of the method in handling situations that have traditionally been problematic for structure alignment methods. Despite the good performance with the AFPRMSD distance metric, one must bear in mind that such metrics are not optimized by the topological alignment and that this method is a distinctly different from distance-based alignment methods.

In addition to the versatility of handling pairwise alignments, the topological alignment method can easily be extended to areas of structural bioinformatics that have traditionally been very difficult because of their computational intensity. Two of these include fast database searching and multiple structure alignment. Our results using TCLUSTALW are particularly encouraging with the example of the alignment of TPK family members. Members of the TPK family all contain a Universal Core Domain consisting of a small, mostly β-sheeted N-terminal subdomain and a larger mostly α-helical C-terminal subdomain [20]. Within this Core Domain are the regions responsible for kinase activation, ATP binding, and phosphotransfer reaction. While members of the superfamily have undergone numerous evolutionary modifications and have low sequence similarities, they do share several key conserved residues. These residues allow the proteins to maintain a structurally well-conserved catalytic core critical for functional activity. The Universal Core Domain of the TPKs is comprised of 9 major β-strands labeled 1–9 and 9 major α-helices labeled A-I. Scheeff and Bourne identified 24 alignment positions whose residues are highly conserved or exhibit extremely similar chemical-physical properties [18]. Thirteen of these positions are conserved as hydrophobic residues, which serve key roles in maintaining the structural network of the protein kinase. Another 8 residues are charged, participating as ionic members with other residues in other strands, which help in stabilizing the orientation and configuration of the subdomains. As seen in Table 4, our unsupervised method gives results that are strikingly similar to the hand alignment constructed from biological intuition.

## Conclusion

This work shows initial encouraging results for developing a suite of structure alignment software tools based on a geometric encoding of protein structures. With a limited exploration of the parameters of the method, competitive performance of pairwise alignment has been demonstrated. Additionally, a computationally efficient and accurate multiple structure alignment has been achieved. The advantage of this method over other approaches is that it performs alignments on a well-defined length scale as dictated by the sliding window employed in generating the geometric alphabet. Current work is extending the method to rapid database searching using SBLAST, the structural equivalent of BLAST. Additional work will also focus on developing a range of substitution matrices based on different block and evolutionary models. Also, a more systematic exploration of alphabet size and segment size is currently underway. Thus, there is significant opportunity to further optimize this unique set of structural alignment software tools.

## Methods

### Calculating the writhing number

**Figure 1:**
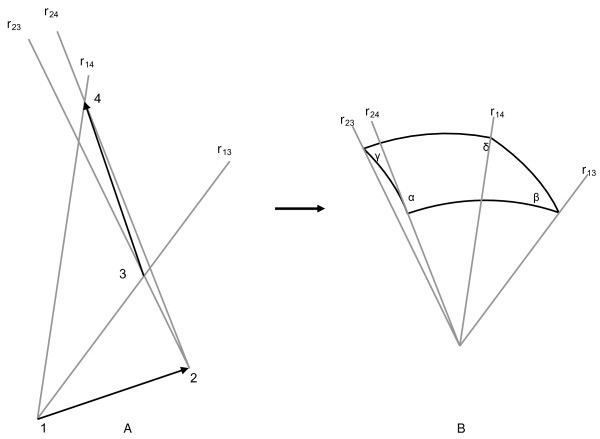
**Definition of vectors for a polygonal curve**. Definition of vectors used in the computation of the writhe number of two segments of a polygonal curve. Points 1 and 2 define the first segment, and 3 and 4 the second. The vectors **r**_13_, **r**_14_, **r**_23_, and **r**_24_, are translated so that they originate at the center of a unit sphere. The area *A *of the quadrangle they span is given by *A *= α+β+γ+δ-2π, where α is the size of the angle measured in radians.

The writhing number can be calculated for chains of arbitrary length *n *using the experimentally determined three-dimensional coordinates of the α-carbon atoms in the protein chain. These *n *coordinates form a piecewise linear or polygonal curve on *n-1 *edges, which precludes the use of the usual definition of writhing number as an integer valued quantity. The number *n *of α-carbons whose coordinates are used in the calculation is the window size since the writhing number is computed for each consecutive window containing *n *contiguous α-carbons along the protein chain. The technique used for calculating the number is adapted from previous work [21] that calculated the writhing number for an entire polymer chain. The calculation considers the relative orientation of two vectors, requiring four distinct points along the protein chains. The vectors formed from these four points are **r**_13_, **r**_14_, **r**_23_, and **r**_24_, as seen in Figure 1. These vectors are translated so that they originate at the center of a unit sphere. The area of the quadrangle spanned by these vectors is then calculated. Depending on whether the crossing of the original vectors **r**_12 _and **r**_34 _is right or left handed, the writhing number is positive or negative.

To handle polygonal curves with more than four points, the writhing numbers for all the distinct pairs of vectors are added together. Thus, the writhing number *Wr *of a segment of length *N *is given by

Wr=2∑i=1N−3∑j=i+2N−1Ωi,j4π     (2)
 MathType@MTEF@5@5@+=feaafiart1ev1aaatCvAUfKttLearuWrP9MDH5MBPbIqV92AaeXatLxBI9gBaebbnrfifHhDYfgasaacH8akY=wiFfYdH8Gipec8Eeeu0xXdbba9frFj0=OqFfea0dXdd9vqai=hGuQ8kuc9pgc9s8qqaq=dirpe0xb9q8qiLsFr0=vr0=vr0dc8meaabaqaciaacaGaaeqabaqabeGadaaakeaacqWGxbWvdaWgaaWcbaGaemOCaihabeaakiabg2da9iabikdaYmaaqahabaWaaabCaeaadaWcaaqaaiabfM6axnaaBaaaleaacqWGPbqAcqGGSaalcqWGQbGAaeqaaaGcbaGaeGinaqdcciGae8hWdahaaaWcbaGaemOAaOMaeyypa0JaemyAaKMaey4kaSIaeGOmaidabaGaemOta4KaeyOeI0IaeGymaedaniabggHiLdaaleaacqWGPbqAcqGH9aqpcqaIXaqmaeaacqWGobGtcqGHsislcqaIZaWma0GaeyyeIuoakiaaxMaacaWLjaWaaeWaceaacqaIYaGmaiaawIcacaGLPaaaaaa@50DE@

where

Ω_*i*,*j *_= (arcsin(**a**_i,j_·**b**_i,j_) + arcsin(**b**_i,j_·**c**_i,j_) + arcsin(**c**_i,j_·**d**_i,j_) + arcsin(**d**_i,j_·**a**_i,j_))·*sign*(**r**_*j,j*+1 _× **r**_*i,i*+1_·**r**_i,j+1_)     (3)

and

ai,j=ri,j×ri,j+1|ri,j×ri,j+1|,bi,j=ri,j+1×ri+1,j+1|ri,j+1×ri+1,j+1|,ci,j=ri+1,j+1×ri+1,j|ri+1,j+1×ri+1,j|,di,j=ri+1,j×ri,j|ri+1,j×ri,j|     (4)
 MathType@MTEF@5@5@+=feaafiart1ev1aaatCvAUfKttLearuWrP9MDH5MBPbIqV92AaeXatLxBI9gBaebbnrfifHhDYfgasaacH8akY=wiFfYdH8Gipec8Eeeu0xXdbba9frFj0=OqFfea0dXdd9vqai=hGuQ8kuc9pgc9s8qqaq=dirpe0xb9q8qiLsFr0=vr0=vr0dc8meaabaqaciaacaGaaeqabaqabeGadaaakeaafaqabeGacaaabaacbeGae8xyae2aaSbaaSqaaiabdMgaPjabcYcaSiabdQgaQbqabaGccqGH9aqpdaWcaaqaaiab=jhaYnaaBaaaleaacqWGPbqAcqGGSaalcqWGQbGAaeqaaOGaey41aqRae8NCai3aaSbaaSqaaiabdMgaPjabcYcaSiabdQgaQjabgUcaRiabigdaXaqabaaakeaadaabdiqaaiab=jhaYnaaBaaaleaacqWGPbqAcqGGSaalcqWGQbGAaeqaaOGaey41aqRae8NCai3aaSbaaSqaaiabdMgaPjabcYcaSiabdQgaQjabgUcaRiabigdaXaqabaaakiaawEa7caGLiWoaaaGaeiilaWcabaGae8Nyai2aaSbaaSqaaiabdMgaPjabcYcaSiabdQgaQbqabaGccqGH9aqpdaWcaaqaaiab=jhaYnaaBaaaleaacqWGPbqAcqGGSaalcqWGQbGAcqGHRaWkcqaIXaqmaeqaaOGaey41aqRae8NCai3aaSbaaSqaaiabdMgaPjabgUcaRiabigdaXiabcYcaSiabdQgaQjabgUcaRiabigdaXaqabaaakeaadaabdiqaaiab=jhaYnaaBaaaleaacqWGPbqAcqGGSaalcqWGQbGAcqGHRaWkcqaIXaqmaeqaaOGaey41aqRae8NCai3aaSbaaSqaaiabdMgaPjabgUcaRiabigdaXiabcYcaSiabdQgaQjabgUcaRiabigdaXaqabaaakiaawEa7caGLiWoaaaGaeiilaWcabaGae83yam2aaSbaaSqaaiabdMgaPjabcYcaSiabdQgaQbqabaGccqGH9aqpdaWcaaqaaiab=jhaYnaaBaaaleaacqWGPbqAcqGHRaWkcqaIXaqmcqGGSaalcqWGQbGAcqGHRaWkcqaIXaqmaeqaaOGaey41aqRae8NCai3aaSbaaSqaaiabdMgaPjabgUcaRiabigdaXiabcYcaSiabdQgaQbqabaaakeaadaabdiqaaiab=jhaYnaaBaaaleaacqWGPbqAcqGHRaWkcqaIXaqmcqGGSaalcqWGQbGAcqGHRaWkcqaIXaqmaeqaaOGaey41aqRae8NCai3aaSbaaSqaaiabdMgaPjabgUcaRiabigdaXiabcYcaSiabdQgaQbqabaaakiaawEa7caGLiWoaaaGaeiilaWcabaGae8hzaq2aaSbaaSqaaiabdMgaPjabcYcaSiabdQgaQbqabaGccqGH9aqpdaWcaaqaaiab=jhaYnaaBaaaleaacqWGPbqAcqGHRaWkcqaIXaqmcqGGSaalcqWGQbGAaeqaaOGaey41aqRae8NCai3aaSbaaSqaaiabdMgaPjabcYcaSiabdQgaQbqabaaakeaadaabdiqaaiab=jhaYnaaBaaaleaacqWGPbqAcqGHRaWkcqaIXaqmcqGGSaalcqWGQbGAaeqaaOGaey41aqRae8NCai3aaSbaaSqaaiabdMgaPjabcYcaSiabdQgaQbqabaaakiaawEa7caGLiWoaaaaaaiaaxMaacaWLjaWaaeWaceaacqaI0aanaiaawIcacaGLPaaaaaa@D9BB@

with **r**_*i*,*i*+1 _representing the vector between points *i *and *i+1*. The double summation is over all pairs of vectors with no common points and *N *is the number of points in the polygonal segment. In this work, segments (or window sizes) of 4, 5, 6 or 10 points (α-carbon atoms) were investigated. The right- or left-handedness of the crossing of the two segments is determined by sign ((**r**_*j*,*j*+1 _× **r**_*i*,*i*+1_)·**r**_*i*,*j*+1_). Larger positive or negative values of *Wr *indicate a greater degree of the curvature.

### Defining an alphabet for the geometric measure

**Figure 2:**
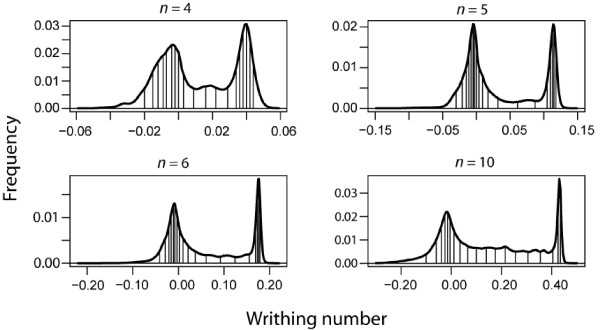
**Distribution of writhing numbers across protein structures**. The distribution of writhing numbers from segments of all proteins in the PDB using a window size of 4, 5, 6 and 10. The histrogram was broken up into twenty regions of constant population (area under the curve). These 20 regions were used to define a topological alphabet. Notice that the range of writhing number increases with segment size.

Using Equation 2, the writhing number for each window was calculated for all PDB proteins available from the RCSB Data Bank. The frequency of occurrence of writhing numbers calculated using a sliding window of 4, 5, 6 and 10 residues is shown in Figure 2 for the entire PDB. The magnitude of the writhing numbers varied according to window size as larger windows can sustain larger writhes. Analysis of different classes of proteins reveals that the large peak with positive writhing numbers in the histogram is due predominantly to α-helical regions while the peak with negative numbers is predominantly a result of β-sheet structures. The writhing numbers were encoded in a 20 letter alphabet by partitioning the histogram into 20 bins and assigning a letter to each bin. For example, with a window size of 5 the letter A represented writhing numbers between -0.05 and -0.03, the letter B represented writhing numbers between -0.03 and -0.021, etc. The histograms in Figure 2 was partitioned into twenty segments in such a manner that the area under the curve of each bin was equal, ensuring that each letter represents the same fraction of total number of observed writhing numbers. This manner of partitioning maximizes the information content *I *of the alphabet. Using Shannon's definition of information entropy, *I*

I=−∑ipilog⁡2pi     (5)
 MathType@MTEF@5@5@+=feaafiart1ev1aaatCvAUfKttLearuWrP9MDH5MBPbIqV92AaeXatLxBI9gBaebbnrfifHhDYfgasaacH8akY=wiFfYdH8Gipec8Eeeu0xXdbba9frFj0=OqFfea0dXdd9vqai=hGuQ8kuc9pgc9s8qqaq=dirpe0xb9q8qiLsFr0=vr0=vr0dc8meaabaqaciaacaGaaeqabaqabeGadaaakeaacqWGjbqscqGH9aqpcqGHsisldaaeqbqaaiabdchaWnaaBaaaleaacqWGPbqAaeqaaOGagiiBaWMaei4Ba8Maei4zaC2aaSbaaSqaaiabikdaYaqabaGccqWGWbaCdaWgaaWcbaGaemyAaKgabeaaaeaacqWGPbqAaeqaniabggHiLdGccaWLjaGaaCzcamaabmGabaGaeGynaudacaGLOaGaayzkaaaaaa@422E@

the entropy function is maximized when *p*_*i *_= *p*_*j*_, where *p*_*i *_is the probability that an arbitrary writhing number is assigned to the *i*^th ^letter of the alphabet. Using the writhing number bins and their corresponding letters, all PDB proteins were encoded into the "geometric alphabet". The encoding of writhing numbers into a geometric alphabet ignores the identity of the amino acids themselves.

### Calculating a block substitution matrix

A substitution matrix was calculated to score alphabet substitutions when comparing proteins structures encoded by the geometric alphabet. This matrix is referred to as TBLOSUM and was determined from multiple sequence alignments of closely related proteins found in the PDB. Using their SCOP classification, 44,234 proteins were grouped into 589 families as defined by their SCOP lineage (a list of these families can be obtained upon request to the authors). Only those families consisting of more than 20 members were considered. These proteins were aligned using CLUSTALW based on their original amino acid sequences using the default BLOSUM62 matrix, a gap opening penalty of 4 and a gap extension penalty of 1. Following the alignment, the geometric alphabet was superimposed upon the sequence. The statistics of geometric alphabet substitutions were determined for alignment blocks. The transitional frequencies for all possible transitions are given as:

fij={i≠j,fi×fji=j,fi(fi−1)2     (6)
 MathType@MTEF@5@5@+=feaafiart1ev1aaatCvAUfKttLearuWrP9MDH5MBPbIqV92AaeXatLxBI9gBaebbnrfifHhDYfgasaacH8akY=wiFfYdH8Gipec8Eeeu0xXdbba9frFj0=OqFfea0dXdd9vqai=hGuQ8kuc9pgc9s8qqaq=dirpe0xb9q8qiLsFr0=vr0=vr0dc8meaabaqaciaacaGaaeqabaqabeGadaaakeaacqWGMbGzdaWgaaWcbaGaemyAaKMaemOAaOgabeaakiabg2da9maaceqabaqbaeaabiqaaaqaaiabdMgaPjabgcMi5kabdQgaQjabcYcaSiabdAgaMnaaBaaaleaacqWGPbqAaeqaaOGaey41aqRaemOzay2aaSbaaSqaaiabdQgaQbqabaaakeaacqWGPbqAcqGH9aqpcqWGQbGAcqGGSaaldaWcaaqaaiabdAgaMnaaBaaaleaacqWGPbqAaeqaaOWaaeWaceaacqWGMbGzdaWgaaWcbaGaemyAaKgabeaakiabgkHiTiabigdaXaGaayjkaiaawMcaaaqaaiabikdaYaaaaaaacaGL7baacaWLjaGaaCzcamaabmGabaGaeGOnaydacaGLOaGaayzkaaaaaa@52FD@

These transition frequencies for each amino acid pair are summed across all blocks for all aligned families. The frequency of members of the alphabet is obtained by simply summing over respective transition frequencies. These single alphabet frequencies are used to calculate the expected number of transition frequencies, e_XY_, assuming that alphabet pairs, X, Y, occur randomly with the members of a pair being proportional to their respective alphabet frequency. The score for any transition is the negative log-ratio of the observed frequency of the transition to the expected frequency of the transition, derived in the same manner of a BLOSUM substitution matrix:

Sij=−log⁡(fijeij)     (7)
 MathType@MTEF@5@5@+=feaafiart1ev1aaatCvAUfKttLearuWrP9MDH5MBPbIqV92AaeXatLxBI9gBaebbnrfifHhDYfgasaacH8akY=wiFfYdH8Gipec8Eeeu0xXdbba9frFj0=OqFfea0dXdd9vqai=hGuQ8kuc9pgc9s8qqaq=dirpe0xb9q8qiLsFr0=vr0=vr0dc8meaabaqaciaacaGaaeqabaqabeGadaaakeaacqWGtbWudaWgaaWcbaGaemyAaKMaemOAaOgabeaakiabg2da9iabgkHiTiGbcYgaSjabc+gaVjabcEgaNnaabmGabaWaaSaaaeaacqWGMbGzdaWgaaWcbaGaemyAaKMaemOAaOgabeaaaOqaaiabdwgaLnaaBaaaleaacqWGPbqAcqWGQbGAaeqaaaaaaOGaayjkaiaawMcaaiaaxMaacaWLjaWaaeWaceaacqaI3aWnaiaawIcacaGLPaaaaaa@44C4@

**Figure 3:**
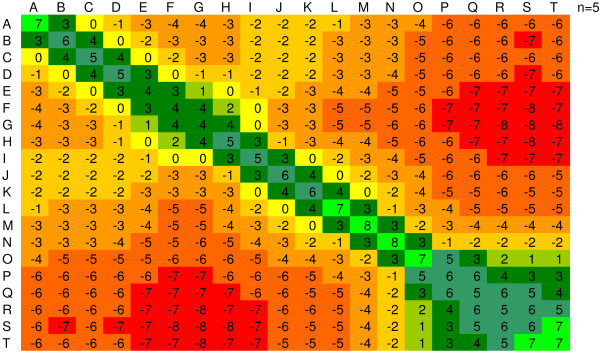
**The BLOCK scoring matrix for the encoded writhing number**. Diagram represents a color coded scoring matrix for an alphabet of 20 letters and a window size of 5.

If the transition between i and j occur more frequently than random, it is given a negative score. However, if the transition occurs less frequently than random, the transition is assigned a positive value. Figure 3 shows the Scoring Matrix for a bin size of 5 when all PDB proteins are considered. TBLOSUM matrices were calculated for each window size (4, 5, 6 and 10) based on the same protein alignment but the different alphabet assignments derived from the histograms in Figure 2.

### Calculating RMSD scores for alignments

Pairwise geometric alignments using the local dynamic programming algorithm TLOCAL optimize the alignment score based on the new scoring system (TBLOSUM). As previously noted, this approach to protein alignment is not intended to minimize global RMSD. Rather, it aligns regions of the proteins that show similarity in local topological profiles and does not allow a direct Cartesian rendering of the alignment. To allow for a comparison of our method with other alignment techniques, we sought a simple way to compute a RMSD for an alignment based on the topological alignment. We used the topological alignment to identify aligned fragment pairs (AFPs). The RMSD of the AFPs are computed for any pair containing at least four aligned pairs. As an example, we consider the following:

VNLDW--Q-QWTW

TPLDWOPQRRWSY

For the five pairs making up the first AFP and the four pairs in the third AFP, we compute a composite RMSD score, but for the single pair of Qs in the middle, no RMSD can be computed and these are not considered in our AFP RMSD calculation. The RMSD values for the AFPs are calculated using the UCSF Chimera package from the Resource for Biocomputing, Visualization, and Informatics at the University of California, San Francisco, which performs rigid translations and rotations to minimize the RMSD between the aligned residues of a pairwise alignment. We calculate RMSD for each AFP. The score for each block is squared and multiplied by its length in aligned pairs. The resulting numbers are summed and divided by the total number of aligned pairs in all the AFPs used. The square root of this number is taken as RMSD of the alignment. This procedure was applied to the CE alignments, as well as the TLOCAL alignments. One must bear in mind that CE was not designed to minimize the RMSD calculated in this fashion and is not optimized for this scoring function.

### Performing the alignments

All alignments were performed using open source versions of the Smith-Waterman dynamic programming algorithm and CLUSTALW. The run time for these applications do not differ from those found in sequence alignment applications. The computationally intense aspect of the work is the encoding of the PDB coordinates into a library of geometric sequences. At the time of this work, the library consisted of 52,087 proteins with a database length of 15,072,799 amino acids. For a window size of 4, cpu run time was 4.71 hours. For a window size of 10, the run time was 98.76 hours. All results were obtained on an IRIX64 server with 16 CPUs with 14G available memory and 128 M swap.

## Authors' contributions

AR worked on pairwise alignments and comparison to other methods. PC worked on multiple structure alignments. AR and PC worked on coding of writhe numbers and PC worked on libraries. GD provided the original impetus for the project and oversaw the project.

## Supplementary Material

Additional File 1 Comparison of topological alignments for different window sizes for a group of "difficult" proteins. Click here for file

Additional File 2 Comparison of different alignment methods for "difficult" proteins. Click here for file

Additional File 3 Comparison of the effect on window size (TLOCAL) and alignment method on alignment scores for hinged proteins. Click here for file

Additional File 4 Multiple sequence alignment of the kinase superfamily - Comparison of topological MSA with hand MSA Click here for file
