# Supplementary material for: Structure alignment based on coding of local geometric measures
Source: BMC Bioinformatics. 2006 Jul 14;7:346. doi: 10.1186/1471-2105-7-346 (PMC1559724; doi:10.1186/1471-2105-7-346)
Supplement: Additional File 1 — Comparison of topological alignments for different window sizes for a group of "difficult" proteins. [file 1471-2105-7-346-S1.doc]

Table 1. Comparison of topological alignments for different window sizes for a group of “difficult” proteins.

|  | *n* = 4 | | | | *n* = 5 | | | |
| --- | --- | --- | --- | --- | --- | --- | --- | --- |
|  | # aligned | score | AFPRMSD (Å) | Reduced  AFPRMSD•10 ^3^ | # aligned | score | AFPRMSD (Å) | Reduced  AFPRMSD•10 ^3^ |
| 1TEN - 3HHR:B | 59 | 221 | 1.535 | 2.315 | 71 | 180 | 1.315 | 6.174 |
| 1BGE:B - 2GMF:A | 65 | 230 | 1.196 | 1.733 | 73 | 231 | 0.777 | 3.548 |
| 1CEW:I - 1MOL:A | 69 | 221 | 1.470 | 2.217 | 67 | 173 | 1.180 | 5.871 |
| 1CID - 2RHE | 73 | 165 | 1.939 | 3.917 | 70 | 151 | 1.516 | 7.219 |
| 1CRL - 1EDE | 182 | 580 | 1.897 | 1.090 | 197 | 615 | 1.214 | 2.054 |
| 1FXI:A - 1UBQ | 48 | 146 | 1.571 | 3.587 | 59 | 98 | 1.448 | 8.181 |
| 1TIE - 4FGF | 50 | 183 | 0.696 | 1.268 | 73 | 184 | 1.827 | 8.342 |
| 2AZA:A - 1PAZ | 67 | 162 | 1.304 | 2.683 | 64 | 122 | 1.316 | 6.854 |
| 2SIM - 1NSB:A | 197 | 513 | 1.505 | 0.978 | 179 | 457 | 1.669 | 3.108 |
| 3HLA:B - 2RHE | 36 | 150 | 1.461 | 3.247 | 72 | 140 | 2.213 | 10.245 |
|  |  |  |  | |  |  |  |  |
|  | *n* = 6 | | | | *n* = 10 | | | |
|  | # aligned | score | AFRMSD (Å) | Reduced  AFPRMSD•10 ^3^ | # aligned | score | AFPRMSD (Å) | Reduced  AFPRMSD•10 ^3^ |
| 1TEN - 3HHR:B | 55 | 201 | 0.976 | 1.619 | 75 | 111 | 1.642 | 7.298 |
| 1BGE:B - 2GMF:A | 79 | 285 | 0.589 | 0.689 | 89 | 254 | 2.942 | 11.019 |
| 1CEW:I - 1MOL:A | 79 | 188 | 1.732 | 3.071 | 80 | 188 | 1.843 | 7.679 |
| 1CID - 2RHE | 75 | 154 | 1.782 | 3.857 | 82 | 168 | 2.259 | 9.183 |
| 1CRL - 1EDE | 194 | 663 | 1.924 | 0.967 | 227 | 571 | 2.642 | 3.880 |
| 1FXI:A - 1UBQ | 54 | 156 | 1.493 | 3.190 | 52 | 94 | 2.488 | 15.949 |
| 1TIE - 4FGF | 82 | 217 | 2.231 | 3.427 | 87 | 128 | 2.417 | 9.261 |
| 2AZA:A - 1PAZ | 66 | 134 | 1.582 | 3.935 | 78 | 126 | 3.629 | 15.509 |
| 2SIM - 1NSB:A | 188 | 449 | 2.526 | 1.875 | 256 | 324 | 4.164 | 05.422 |
| 3HLA:B - 2RHE | 69 | 175 | 1.754 | 3.341 | 80 | 129 | 2.079 | 8.663 |
