# Supplementary material for: Structure alignment based on coding of local geometric measures
Source: BMC Bioinformatics. 2006 Jul 14;7:346. doi: 10.1186/1471-2105-7-346 (PMC1559724; doi:10.1186/1471-2105-7-346)
Supplement: Additional File 2 — Comparison of different alignment methods for "difficult" proteins. [file 1471-2105-7-346-S2.doc]

Table 2. Comparison of different alignment methods for “difficult” proteins .

|  | *n* = 5 | | | FATCAT | | | CE | | |
| --- | --- | --- | --- | --- | --- | --- | --- | --- | --- |
|  | # aligned | AFPRMSD (Å) | Reduced  AFPRMSD•10 ^3^ | # aligned | AFPRMSD  (Å) | Reduced  AFPRMSD•10 ^3^ | # aligned | AFPRMSD  (Å) | Reduced  AFPRMSD•10 ^3^ |
| 1TEN - 3HHR:B | 71 | 1.315 | 6.174 | 87 | 1.900 | 7.280 | 87 | 1.400 | 5.364 |
| 1BGE:B - 2GMF:A | 73 | 0.777 | 3.548 | 100 | 3.190 | 10.633 | 100 | 2.065 | 6.883 |
| 1CEW:I - 1MOL:A | 67 | 1.180 | 5.871 | 83 | 2.440 | 9.799 | 81 | 1.665 | 6.852 |
| 1CID - 2RHE | 70 | 1.516 | 7.219 | 100 | 3.110 | 10.367 | 96 | 2.288 | 7.944 |
| 1CRL - 1EDE | 197 | 1.214 | 2.054 | 269 | 3.550 | 4.399 | 213 | 2.377 | 3.720 |
| 1FXI:A - 1UBQ | 59 | 1.448 | 8.181 | 63 | 3.010 | 15.926 | 64 | 2.203 | 11.474 |
| 1TIE - 4FGF | 73 | 1.827 | 8.342 | 117 | 3.050 | 8.689 | 113 | 1.875 | 5.531 |
| 2AZA:A - 1PAZ | 64 | 1.316 | 6.854 | 87 | 3.010 | 11.533 | 78 | 1.903 | 8.132 |
| 2SIM - 1NSB:A | 179 | 1.669 | 3.108 | 286 | 3.070 | 3.578 | 268 | 1.937 | 2.242 |
| 3HLA:B - 2RHE | 72 | 2.213 | 10.245 | 79 | 2.810 | 11.857 | 84 | 2.520 | 10.000 |
