# Supplementary material for: Structure alignment based on coding of local geometric measures
Source: BMC Bioinformatics. 2006 Jul 14;7:346. doi: 10.1186/1471-2105-7-346 (PMC1559724; doi:10.1186/1471-2105-7-346)
Supplement: Additional File 3 — Comparison of the effect on window size (TLOCAL) and alignment method on alignment scores for hinged proteins. [file 1471-2105-7-346-S3.doc]

Table 3. Comparison of the effect on window size (TLOCAL) and alignment method on alignment scores for hinged proteins.

|  | *n* = 4 | | | | | *n* = 5 | | | | | | |  |
| --- | --- | --- | --- | --- | --- | --- | --- | --- | --- | --- | --- | --- | --- |
|  | # aligned | | Topological  score | AFPRMSD  (Å) | Reduced  AFPRMSD  •10 ^3^ | # aligned | Topological  score | AFPRMSD  (Å) | | Reduced  AFPRMSD  •10 ^3^ | | |  |
| 1ADJ:A - 1QF6:A | 272 | | 980 | 1.189 | 0.404 | 315 | 881 | 1.258 | | 1.331 | | |  |
| 1AKE:A - 2AK3:A | 188 | | 819 | 3.756 | 1.529 | 196 | 778 | 1.752 | | 2.980 | | |  |
| 1CLL - 2BBM:A | 121 | | 526 | 1.102 | 0.698 | 129 | 487 | 1.313 | | 3.393 | | |  |
| 1DAN:L - 1B9W:A | 51 | | 183 | 2.167 | 3.947 | 33 | 138 | 2.800 | | 28.283 | | |  |
| 1GGG:A - 1HPB:P | 177 | | 684 | 1.729 | 0.843 | 191 | 689 | 1.017 | | 1.775 | | |  |
| 1LFG - 1LFH | 675 | | 3389 | 3.653 | 0.359 | 683 | 3415 | 5.033 | | 2.456 | | |  |
| 1QCF:A - 1FMK | 394 | | 1922 | 1.308 | 0.227 | 404 | 1773 | 1.363 | | 1.125 | | |  |
| 1TCR:B - 1MCP:L | 157 | | 543 | 1.321 | 0.811 | 174 | 515 | 1.211 | | 2.320 | | |  |
| 1TFD - 1LFH | 241 | | 998 | 2.382 | 0.796 | 254 | 1025 | 2.222 | | 2.916 | | |  |
| 1TKI:A - 1FMK | 195 | | 718 | 1.497 | 0.695 | 193 | 686 | 1.212 | | 2.093 | | |  |
| 1TOP - 2BBM:A | 126 | | 516 | 1.331 | 0.860 | 137 | 474 | 3.645 | | 8.869 | | |  |
| 1UKE - 2AK3:A | 155 | | 649 | 0.800 | 0.411 | 157 | 623 | 1.153 | | 2.448 | | |  |
| 1WDN:A - 1GGG:A | 209 | | 924 | 3.462 | 1.249 | 214 | 899 | 4.634 | | 7.218 | | |  |
| 2CLR:A - 3FRU:A | 240 | | 885 | 1.792 | 0.675 | 234 | 835 | 1.766 | | 2.516 | | |  |
| 4FAB:L - 1MCP:L | 183 | | 657 | 1.626 | 0.825 | 209 | 668 | 3.400 | | 5.423 | | |  |
| 1AJ3 - 2SPC:A | 72 | | 351 | 0.825 | 0.783 | 85 | 425 | 0.781 | | 3.063 | | |  |
|  |  | |  |  |  |  |  |  | |  | | |  |
|  |  |  | |  |  |  |  |  |  | |  |  | |
|  |  | *n* = 6 | |  |  |  | *n* = 10 |  |  | | FATCAT |  | |
|  | # aligned | Topological  score | | AFPRMSD (Å) | Reduced  AFPRMSD  •10 ^3^ | # aligned | Topological  score | AFPRMSD (Å) | Reduced  AFPRMSD  •10 ^3^ | | AFPRMSD  (Å) | Reduced  AFPRMSD  •10 ^3^ | |
| 1ADJ:A - 1QF6:A | 304 | 1059 | | 1.509 | 0.475 | 356 | 1004 | 2.613 | 2.447 | | 2.680 | 2.545 | |
| 1AKE:A - 2AK3:A | 201 | 870 | | 3.336 | 1.278 | 187 | 811 | 1.088 | 1.939 | | 1.540 | 2.541 | |
| 1CLL - 2BBM:A | 130 | 592 | | 1.375 | 0.774 | 130 | 560 | 1.385 | 3.551 | | 2.280 | 5.278 | |
| 1DAN:L - 1B9W:A | 51 | 179 | | 2.220 | 4.134 | 70 | 157 | 3.073 | 14.633 | | 2.390 | 9.958 | |
| 1GGG:A - 1HPB:P | 196 | 767 | | 1.349 | 0.586 | 200 | 762 | 1.705 | 2.842 | | 1.590 | 2.488 | |
| 1LFG - 1LFH | 683 | 3547 | | 4.125 | 0.388 | 684 | 3343 | 4.110 | 2.003 | | 0.890 | 0.432 | |
| 1QCF:A - 1FMK | 417 | 1858 | | 1.384 | 0.248 | 410 | 1745 | 1.569 | 1.276 | | 2.270 | 1.747 | |
| 1TCR:B - 1MCP:L | 182 | 525 | | 1.517 | 0.963 | 190 | 480 | 2.740 | 4.807 | | 2.200 | 3.443 | |
| 1TFD - 1LFH | 267 | 1096 | | 2.811 | 0.855 | 270 | 1033 | 3.173 | 3.917 | | 1.370 | 1.575 | |
| 1TKI:A - 1FMK | 205 | 769 | | 1.360 | 0.59 | 228 | 762 | 2.616 | 3.825 | | 3.070 | 4.300 | |
| 1TOP - 2BBM:A | 123 | 563 | | 3.498 | 2.071 | 124 | 512 | 1.632 | 4.387 | | 2.280 | 5.241 | |
| 1UKE - 2AK3:A | 167 | 695 | | 1.464 | 0.702 | 166 | 689 | 1.486 | 2.984 | | 2.970 | 5.266 | |
| 1WDN:A - 1GGG:A | 107 | 1014 | | 5.023 | 1.651 | 210 | 978 | 3.192 | 5.067 | | 1.010 | 1.530 | |
| 2CLR:A - 3FRU:A | 245 | 946 | | 2.483 | 0.875 | 250 | 855 | 2.495 | 3.327 | | 3.060 | 4.163 | |
| 4FAB:L - 1MCP:L | 204 | 726 | | 1.178 | .541 | 212 | 648 | 3.891 | 6.118 | | 1.400 | 2.151 | |
| 1AJ3 - 2SPC:A | 79 | 417 | | 1.529 | 1.222 | 75 | 387 | 1.154 | 5.129 | | 2.020 | 7.240 | |
|  |  |  | |  |  |  |  |  |  | |  |  | |
