# Supplementary material for: Structure alignment based on coding of local geometric measures
Source: BMC Bioinformatics. 2006 Jul 14;7:346. doi: 10.1186/1471-2105-7-346 (PMC1559724; doi:10.1186/1471-2105-7-346)
Supplement: Additional File 4 — Multiple sequence alignment of the kinase superfamily - Comparison of topological MSA with hand MSA [file 1471-2105-7-346-S4.doc]

Table 4: Multiple Sequence Alignment of the Kinase Superfamily - Comparison of Topological MSA with Hand MSA

1CDK:A FERI-KTLGTGSFGRVMLVKHKE-----TGNHFAMKILDKQK------VVKLKQIE-HTL 1CDK:A -FERIKTLGTGSFGRVMLVKHKE-----TGNHFAMKILDKQ-------KVVKLKQIEHTL

1O6L:A FDYL-KLLGKGTFGKVILVREKA-----TGRYYAMKILRKEV------IIAKDEVA-HTV 1O6L:A -FDYLKLLGKGTFGKVILVREKA-----TGRYYAMKILRKE-------VIIAKDEVAHTV

1OMW:A FSVH-RIIGRGGFGEVYGCRKAD-----TGKMYAMKCLDKKR------IKMKQGET-LAL 1OMW:A -FSVHRIIGRGGFGEVYGCRKAD-----TGKMYAMKCLDKKR-----IKMKQGETLALNE

1H1W:A FKFG-KILGEGSFSTVVLARELA-----TSREYAIKILEKRH------IIKENKVP-YVT 1H1W:A -FKFGKILGEGSFSTVVLARELA-----TSREYAIKILEKR-------HIIKENKVPYVT

1MUO:A FEIG-RPLGKGKFGNVYLAREKQ-----SKFILALKVLFKAQ------LEKAGVEH-QLR 1MUO:A -FEIGRPLGKGKFGNVYLAREKQ-----SKFILALKVLFKAQ-------LEKAGVEHQLR

1TKI:A YMIA-EDLGRGEFGIVHRCVETS-----SKKTYMAKFVKVKG----------TDQV-LVK 1TKI:A -YMIAEDLGRGEFGIVHRCVETS-----SKKTYMAKFVKVK-----------GTDQVLVK

1JKL:A YDTG-EELGSGQFAVVKKCREKS-----TGLQYAAKFIKKRR---TKSSRRGVSRE-DIE 1JKL:A -YDTGEELGSGQFAVVKKCREKS-----TGLQYAAKFIKKRRTK----SSRRGVSREDIE

1A06 YDFR-DVLGTGAFSEVILAEDKR-----TQKLVAIKCIAK-------------------E 1A06 -YDFRDVLGTGAFSEVILAEDKR-----TQKLVAIKCIAK-------------------E

1PHK YEPK-EILGRGVSSVVRRCIHKP-----TCKEYAVKIIDVTGGGSFSAEEVQELRE-ATL 1PHK -YEPKEILGRGVSSVVRRCIHKP-----TCKEYAVKIIDVTGGGSFSAEEVQELREATLK

1KWP:A YKVTSQVLGLGINGKVLQIFNKR-----TQEKFALKMLQD--------------CP-KAR 1KWP:A YKVTSQVLGLGINGKVLQIFNKR-----TQEKFALKMLQDCP--------------KARR

1IA8:A WDLV-QTLGEGAYGEVQLAVNRV-----TEEAVAVKIVDMKR-----------CPE-NIK 1IA8:A -WDLVQTLGEGAYGEVQLAVNRV-----TEEAVAVKIVDMK------------RCPENIK

1GNG:A YTDT-KVIGNGSFGVVYQAKLCD-----SGELVAIKKVL-------------QDKR-FKN 1GNG:A -YTDTKVIGNGSFGVVYQAKLCD-----SGELVAIKKVLQ--------------DKRFKN

1HCK FQKV-EKIGEGTYGVVYKARNKL-----TGEVVALKKIR-----------TEGVPS-TAI 1HCK -FQKVEKIGEGTYGVVYKARNKL-----TGEVVALKKIRTE------------GVPSTAI

1JNK YQNL-KPIGSGAQGIVCAAYDAV-----LDRNVAIKKLSRPFQ-------NQTHAK-RAY 1JNK -YQNLKPIGSGAQGIVCAAYDAV-----LDRNVAIKKLSRPF--------QNQTHAKRAY

1HOW:A YILV-RKLGWGHFSTVWLAKDMV-----NNTHVAMKIVRG----------DKVYTE-AAE 1HOW:A -YILVRKLGWGHFSTVWLAKDMV-----NNTHVAMKIVRGDKV------YTEAAEDEIKL

1LP4:A YEVV-RKVGRGKYSEVFEGINVN-----NNEKCIIKILKP------------VKKK-KIK 1LP4:A -YEVVRKVGRGKYSEVFEGINVN-----NNEKCIIKILKPV------------KKKKIKR

1F3M:C YTRF-EKIGQGASGTVYTAMDVA-----TGQEVAIRQMNLQQ---------QPKKE-LII 1F3M:C -YTRFEKIGQGASGTVYTAMDVA-----TGQEVAIRQMNLQ----------QQPKKELII

1O6Y:A YELG-EILGFGGMSEVHLARDLR-----LHRDVAVKVLRADL------ARDPSFYL-RFR 1O6Y:A -YELGEILGFGGMSEVHLARDLR-----LHRDVAVKVLRADLA-------RDPSFYLRFR

1CSN YKVG-RRIGEGSFGVIFEGTNLL-----NNQQVAIKFEPRRS-----------DA-PQLR 1CSN -YKVGRRIGEGSFGVIFEGTNLL-----NNQQVAIKFEPRR-----------SDAPQLRD

1B6C:B IVLQ-ESIGKGRFGEVWRGKW-------RGEEVAVKIFSS------------REER-SWF 1B6C:B -IVLQESIGKGRFGEVWRGKWRG-------EEVAVKIFSSR-------------EERSWF

2SRC LRLE-VKLGQGCFGEVWMGTWNG------TTRVAIKTLKP----------GTMSPE-AFL 2SRC -LRLEVKLGQGCFGEVWMGTWNG------TTRVAIKTLKPGT-----------MSPEAFL

1LUF:A IEYV-RDIGEGAFGRVFQARAPGLLPYEPFTMVAVKMLKEEA--------SADMQA-DFQ 1LUF:A -IEYVRDIGEGAFGRVFQARAPGLLPYEPFTMVAVKMLKEE---------ASADMQADFQ

1IR3:A ITLL-RELGQGSFGMVYEGNARDIIKGEAETRVAVKTVNESA--------SLRERI-EFL 1IR3:A -ITLLRELGQGSFGMVYEGNARDIIKGEAETRVAVKTVNES---------ASLRERIEFL

1M14:A FKKI-KVLGSGAFGTVYKGLWIPEG-EKVKIPVAIKELREAT--------SPKANK-EIL 1M14:A -FKKIKVLGSGAFGTVYKGLWIP-EGEKVKIPVAIKELREA---------TSPKANKEIL

1GJO:A LTLG-KPLGEGCFGQVVMAEAVGI-KPKEAVTVAVKMLKDDA--------TEKDLS-DLV 1GJO:A -LTLGKPLGEGCFGQVVMAEAVG-IKPKEAVTVAVKMLKDD---------ATEKDLSDLV

Strand 1 Strand 2 Strand 3 Strand 1 Strand 2 Strand 3

1CDK:A NEKRILQAVN-----------FPFLVKLEYSFKDN------SNLYMVMEYVPGGEMFSHL 1CDK:A NEKRILQAV------NFPFLVKLEYSFKDNSNLY------MVMEYVPGGEM---FSHLRR

1O6L:A TESRVLQNTR-----------HPFLTALKYAFQTH------DRLCFVMEYANGGELFFHL 1O6L:A TESRVLQNT------RHPFLTALKYAFQTHDRLC------FVMEYANGGEL---FFHLSR

1OMW:A NERIMLSLVSTG--------DCPFIVCMSYAFHTP------DKLSFILDLMNGGDLHYHL 1OMW:A RIMLSLVSTG-----DCPFIVCMSYAFHTPDKLS------FILDLMNGGDL---HYHLSQ

1H1W:A RERDVMSRLD-----------HPFFVKLYFTFQDD------EKLYFGLSYAKNGELLKYI 1H1W:A RERDVMSRL------DHPFFVKLYFTFQDDEKLY------FGLSYAKNGEL---LKYIRK

1MUO:A REVEIQSHLR-----------HPNILRLYGYFHDA------TRVYLILEYAPLGTVYREL 1MUO:A REVEIQSHL------RHPNILRLYGYFHDATRVY------LILEYAPLGTV---YRELQK

1TKI:A KEISILNIAR-----------HRNILHLHESFESM------EELVMIFEFISGLDIFERI 1TKI:A KEISILNIA------RHRNILHLHESFESMEELV------MIFEFISGLDI---FERINT

1JKL:A REVSILKEIQ-----------HPNVITLHEVYENK------TDVILILELVAGGELFDFL 1JKL:A REVSILKEI------QHPNVITLHEVYENKTDVI------LILELVAGGEL---FDFLAE

1A06 NEIAVLHKIK-----------HPNIVALDDIYESG------GHLYLIMQLVSGGELFDRI 1A06 NEIAVLHKI------KHPNIVALDDIYESGGHLY------LIMQLVSGGEL---FDRIVE

1PHK KEVDILRKVSG----------HPNIIQLKDTYETN------TFFFLVFDLMKKGELFDYL 1PHK EVDILRKVS------GHPNIIQLKDTYETNTFFF------LVFDLMKKGEL---FDYLTE

1KWP:A REVELHWRASQ----------CPHIVRIVDVYENLYA--GRKCLLIVMECLDGGELFSRI 1KWP:A EVELHWRAS------QCPHIVRIVDVYENLYAGR--KCLLIVMECLDGGEL---FSRIQD

1IA8:A KEICINKMLN-----------HENVVKFYGHRREG------NIQYLFLEYCSGGELFDRI 1IA8:A KEICINKML------NHENVVKFYGHRREGNIQY------LFLEYCSGGEL---FDRIEP

1GNG:A RELQIMRKLD-----------HCNIVRLRYFFYSSGEKKDEVYLNLVLDYV-PETVYRVA 1GNG:A RELQIMRKL------DHCNIVRLRYFFYSSGEKKDEVYLNLVLDYVPETVYRVARHYSRA

1HCK REISLLKELN-----------HPNIVKLLDVIHTE------NKLYLVFEFLH-QDLKKFM 1HCK REISLLKEL------NHPNIVKLLDVIHTENKLY------LVFEFLHQDLK---KFMDAS

1JNK RELVLMKCVN-----------HKNIISLLNVFTPQKTLEEFQDVYLVMELMD-ANLCQVI 1JNK RELVLMKCV------NHKNIISLLN-VFTPQKTLEEFQDVYLVMELMDANL---CQVIQM

1HOW:A DEIKLLQRVNDADNTKEDSMGANHILKLLDHFNHKGP--NGVHVVMVFEVL-GENLLALI 1HOW:A LQRVNDADNTKEDSMGANHILKLLDHFNHKGPNG---VHVVMVFEVLGENL---LALIKK

1LP4:A REIKILQNLCG----------GPNIVKLLDIVRDQH----SKTPSLIFEYVNNTDFKVLY 1LP4:A EIKILQNLC------GGPNIVKLLDIVRDQHSKTPS----LIFEYVNNTDF---KVLYPT

1F3M:C NEILVMRENK-----------NPNIVNYLDSYLVG------DELWVVMEYLAGGSLTDVV 1F3M:C NEILVMREN------KNPNIVNYLDSYLVGDELW------VVMEYLAGGSL---TDVVTE

1O6Y:A REAQNAAALN-----------HPAIVAVYDTGEAETP--AGPLPYIVMEYVDGVTLRDIV 1O6Y:A REAQNAAAL------NHPAIVAVYD-TGEAETPA-GPLPYIVMEYVDGVTL---RDIVHT

1CSN DEYRTYKLLAG----------CTGIPNVYYFGQEG------LHNVLVIDLLG-PSLEDLL 1CSN EYRTYKLLA------GCTGIPNVYYFGQEGLHNV-------LVIDLLGPSL---EDLLDL

1B6C:B REAEIYQTVMLR---------HENILGFIAADNKDNG--TWTQLWLVSDYHEHGSLFDYL 1B6C:B REAEIYQTVM----LRHENILGFIAADNKDNGTW--TQLWLVSDYHEHGSL---FDYLNR

2SRC QEAQVMKKLR-----------HEKLVQLYAVVSEE-------PIYIVTEYMSKGSLLDFL 2SRC QEAQVMKKL------RHEKLVQLYAVVSEEPIYI-------VTEYMSKGSL---LDFLKG

1LUF:A REAALMAEFD-----------NPNIVKLLGVCAVG------KPMCLLFEYMAYGDLNEFL 1LUF:A REAALMAEF------DNPNIVKLLGVCAVGKPMC------LLFEYMAYGDLN--EFLRSM

1IR3:A NEASVMKGFT-----------CHHVVRLLGVVSKG------QPTLVVMELMAHGDLKSYL 1IR3:A NEASVMKGF------TCHHVVRLLGVVSKGQPTL------VVMELMAHGDLK--SYLRSL

1M14:A DEAYVMASVD-----------NPHVCRLLGICLTS-------TVQLITQLMPFGCLLDYV 1M14:A DEAYVMASV------DNPHVCRLLGIC-LTSTVQ------LITQLMPFGCLL--DYVREH

1GJO:A SEMEMMKMIGK----------HKNIINLLGACTQD------GPLYVIVEYASKGNLREYL 1GJO:A SEMEMMKMIG-----KHKNIINLLGACTQDGPLY------VIVEYASKGNLR--EYLRAR

Helix C Strand 4 Strand 5 Helix C Strand 4 Strand 5

1CDK:A RRI----------GRFSEPHARFYAAQIVLTFEYLHS--------LDLIYRDLKPENLLI 1CDK:A I---------GRFSEPHARFYAAQIVLTFEYL--------HSLDLIYRDLKPENLLID--

1O6L:A SRE----------RVFTEERARFYGAEIVSALEYLHS--------RDVVYRDIKLENLML 1O6L:A E---------RVFTEERARFYGAEIVSALEYL--------HSRDVVYRDIKLENLMLD--

1OMW:A SQH----------GVFSEADMRFYAAEIILGLEHMHN--------RFVVYRDLKPANILL 1OMW:A H---------GVFSEADMRFYAAEIILGLEHM--------HNRFVVYRDLKPANILLD--

1H1W:A RKI----------GSFDETCTRFYTAEIVSALEYLHG--------KGIIHRDLKPENILL 1H1W:A I---------GSFDETCTRFYTAEIVSALEYL--------HGKGIIHRDLKPENILLN--

1MUO:A QKL----------SKFDEQRTATYITELANALSYCHS--------KRVIHRDIKPENLLL 1MUO:A L---------SKFDEQRTATYITELANALSYC--------HSKRVIHRDIKPENLLLG--

1TKI:A NTS---------AFELNEREIVSYVHQVCEALQFLHS--------HNIGHFDIRPENIIY 1TKI:A S--------AFELNEREIVSYVHQVCEALQFL--------HSHNIGHFDIRPENIIYQT-

1JKL:A AEK----------ESLTEEEATEFLKQILNGVYYLHS--------LQIAHFDLKPENIML 1JKL:A K---------ESLTEEEATEFLKQILNGVYYL--------HSLQIAHFDLKPENIMLLDR

1A06 VEK----------GFYTERDASRLIFQVLDAVKYLHD--------LGIVHRDLKPENLLY 1A06 K---------GFYTERDASRLIFQVLDAVKYL--------HDLGIVHRDLKPENLLYYSL

1PHK TEK----------VTLSEKETRKIMRALLEVICALHK--------LNIVHRDLKPENILL 1PHK K---------VTLSEKETRKIMRALLEVICAL--------HKLNIVHRDLKPENILLD--

1KWP:A QDRGD--------QAFTEREASEIMKSIGEAIQYLHS--------INIAHRDVKPENLLY 1KWP:A R-------GDQAFTEREASEIMKSIGEAIQYL--------HSINIAHRDVKPENLLYTSK

1IA8:A EPD----------IGMPEPDAQRFFHQLMAGVVYLHG--------IGITHRDIKPENLLL 1IA8:A D---------IGMPEPDAQRFFHQLMAGVVYL--------HGIGITHRDIKPENLLLD--

1GNG:A RHYSRA------KQTLPVIYVKLYMYQLFRSLAYIHS--------FGICHRDIKPQNLLL 1GNG:A K---------QTLPVIYVKLYMYQLFRSLAYI--------HSFGICHRDIKPQNLLLD--

1HCK DASAL--------TGIPLPLIKSYLFQLLQGLAFCHS--------HRVLHRDLKPQNLLI 1HCK AL--------TGIPLPLIKSYLFQLLQGLAFC--------HSHRVLHRDLKPQNLLIN--

1JNK QM------------ELDHERMSYLLYQMLCGIKHLHS--------AGIIHRDLKPSNIVV 1JNK -----------ELDHERMSYLLYQMLCGIKHL--------HSAGIIHRDLKPSNIVVK--

1HOW:A KKYEH--------RGIPLIYVKQISKQLLLGLDYMHRR-------CGIIHTDIKPENVLM 1HOW:A YEH-------RGIPLIYVKQISKQLLLGLDYMH-------RRCGIIHTDIKPENVLMEIV

1LP4:A -------------PTLTDYDIRYYIYELLKALDYCHS--------QGIMHRDVKPHNVMI 1LP4:A L------------TDYDIRYYIYELLKALDYC--------HSQGIMHRDVKPHNVMIDH-

1F3M:C TET-----------CMDEGQIAAVCRECLQALEFLHS--------NQVIHRDIKSDNILL 1F3M:C T----------CMDEGQIAAVCRECLQALEFL--------HSNQVIHRDIKSDNILLG--

1O6Y:A HTE----------GPMTPKRAIEVIADACQALNFSHQ--------NGIIHRDVKPANIMI 1O6Y:A E---------GPMTPKRAIEVIADACQALNFS--------HQNGIIHRDVKPANIMIS--

1CSN DLCG---------RKFSVKTVAMAAKQMLARVQSIHE--------KSLVYRDIKPDNFLI 1CSN C--------GRKFSVKTVAMAAKQMLARVQSI--------HEKSLVYRDIKPDNFLIGRP

1B6C:B NRY-----------TVTVEGMIKLALSTASGLAHLHMEIVGTQGKPAIAHRDLKSKNILV 1B6C:B Y----------TVTVEGMIKLALSTASGLAHLHMEIVGTQGKPAIAHRDLKSKNILVK--

2SRC KGETG--------KYLRLPQLVDMAAQIASGMAYVER--------MNYVHRDLRAANILV 2SRC ETG-------KYLRLPQLVDMAAQIASGMAYV--------ERMNYVHRDLRAANILVG--

1LUF:A RSMSP--------PPLSCAEQLCIARQVAAGMAYLSE--------RKFVHRDLATRNCLV 1LUF:A SPP--------PLSCAEQLCIARQVAAGMAYL--------SERKFVHRDLATRNCLVG--

1IR3:A RSLRPEAENNPGRPPPTLQEMIQMAAEIADGMAYLNA--------KKFVHRDLAARNCMV 1IR3:A RPEAENNPGRPPPTLQEMIQMAAEIADGMAYL--------NAKKFVHRDLAARNCMVA--

1M14:A REHK---------DNIGSQYLLNWCVQIAKGMNYLED--------RRLVHRDLAARNVLV 1M14:A KDN---------IGSQYLLNWCVQIAKGMNYL--------EDRRLVHRDLAARNVLVK--

1GJO:A RARR---------EQMTFKDLVSCTYQLARGMEYLAS--------QKCIHRDLAARNVLV 1GJO:A R---------EQMTFKDLVSCTYQLARGMEYL--------ASQKCIHRDLAARNVLVT--

Helix E Strand 6 Strand 7 Helix E Strand 6 Strand 7

1CDK:A DQ------QGYIQVTDFGFAKRVK------GRTWT----LCGTPEYLAPEIILS------ 1CDK:A --QQGYI--QVTDFGFA---KR-----VKGRTWTL--CGTPEYLAPEII------LSKGY

1O6L:A DK------DGHIKITDFGLCKEGIS---DGATM----K-FCGTPEYLAPEVLED------ 1O6L:A --KDGHI--KITDFGLC---KE-----GISDGATMKFCGTPEYLAPEVL------EDNDY

1OMW:A DE------HGHVRISDLGLACDFSK-----KKP----HASVGTHGYMAPEVLQK-----G 1OMW:A --EHGHV--RISDLGLAC---------DFSKKKPHASVGTHGYMAPEVLQ-----KGVAY

1H1W:A NE------DMHIQITDFGTAKVLSP------ARAN----FVGTAQYVSPELLTE------ 1H1W:A --EDMHI--QITDFGTA----------KVLSPARANFVGTAQYVSPELLT------EKSA

1MUO:A GS------AGELKIADFGWS-------------------LCGTLDYLPPEMIEG------ 1MUO:A --SAGEL--KIADFGWS-------------------LCGTLDYLPPEMIE------GRMH

1TKI:A QTR----RSSTIKIIEFGQARQLKP-----GDN---FRLLFTAPEYYAPEVHQH------ 1TKI:A -RRSSTI--KIIEFGQA---RQ-----LKPGDNFRLLFTAPEYYAPEVHQ------HDVV

1JKL:A LDRN--VPKPRIKIIDFGLAHKID------FGNEF--KNIFGTPEFVAPEIVNY------ 1JKL:A NVPKPRI--KIIDFGLA---HK-----IDFGNEFKNIFGTPEFVAPEIVN------YEPL

1A06 YSLD---EDSKIMISDF--------------------------PGYVAPEVLAQ------ 1A06 DEDSKIM--ISD---------------------------FPGYVAPEVLA------QKPY

1PHK DD------DMNIKLTDFGFSCQLD------PGEKL--REVCGTPSYLAPEIIECSMNDNH 1PHK --DDMNI--KLTDFGFS---CQ-----LDPGEKLREVCGTPSYLAPEIIECSMNDNHPGY

1KWP:A TSKR---PNAIIKLTDFGFAKETT------S---------------------GP------ 1KWP:A --RPNAI-LKLTDFGFAK------------------------ETTSGPEK---------Y

1IA8:A DE------RDNIKISDFGLATVFRYN--NRERLL---NKMCGTLPYVAPELLKR-----R 1IA8:A --ERDNL--KISDFGLATVFRY-----NNRERLLNKMCGTLPYVAPELLK-----RREFH

1GNG:A DPD-----TAVIKLCDFGSAKQLV------RGEPN--VS-ICSRYYRAPELIFG-----A 1GNG:A --PDTAV--LKLCDFGS---AK-----QLVRGEPNVSICSRYYRAPELIF-----GATDY

1HCK NT------EGAIKLADFGLARAFGVP-----VRTY--THEVVTLWYRAPEILLG-----C 1HCK --TEGAI--KLADFGLARA--F-----GVPVRTYTHEVVTLWYRAPEILL-----GCKYY

1JNK KS------DCTIKILDFGLA-----------SFMM--TPYVVTRYYRAPEVILG------ 1JNK --SDCTL--KILDFGLA-------------SFMMTPYVVTRYYRAPEVIL------GMGY

1HOW:A EIVDSPENLIQIKIADLGNACWYD--------EHY--TNSIQTREYRSPEVLLG------ 1HOW:A DSPENLIQIKIADLGNAC----------WYDEHYTNSIQTREYRSPEVLL------GAPW

1LP4:A DHE-----LRKIRLIDWGLAEFYH------PGKEY--NVRVASRYFKGPELLVD-----L 1LP4:A --ELRKL--RLIDWGLAE---F-----YHPGKEYNVRVASRYFKGPELLVD-----LQDY

1F3M:C GM------DGSVKLTDFGFCAQIT--------------TMVGTPYWMAPEVVTR------ 1F3M:C --MDGSV--KLTDFGFC--------------AQITTMVGTPYWMAPEVVT------RKAY

1O6Y:A SA------TNAVKVMDFGIARAI--------------------AQYLSPEQARG------ 1O6Y:A --ATNAV--KVMDFGI--------------------ARAIAQYLSPEQAR------GDSV

1CSN GRPNS-KNANMIYVVDFGMVKFYRDPVTKQHIPYREKKNLSGTARYMSINTHLG------ 1CSN NSKNANM-IYVVDFGMVKFYRDPVTKQHIPYREKKNLSGTARYMSINTHL------GREQ

1B6C:B KK------NGTCCIADLGLAVRHDSA---TDTIDIAPNHRVGTKRYMAPEVLDD----SI 1B6C:B --KNGTC--CIADLGLAVRHDSA---TDTIDIAPNHRVGTKRYMAPEVLDD---SINMKH

2SRC GE------NLVCKVADFGLARLIEDNE----YTAR--QGAKFPIKWTAPEAALY------ 2SRC --ENLVC--KVADFGLARLIED------NEYTARQGAKFPIKWTAPEAAL-----YGRFT

1LUF:A GE------NMVVKIADFGLSRNIYSADY---YK------DAIPIRWMPPESIFY------ 1LUF:A --ENMVV--KIADFGLSRNIYS-----A----DYYKDAIPIRWMPPESIF-----YNRYT

1IR3:A AH------DFTVKIGDFGMTRDI------ETD--RKGGKGLLPVRWMAPESLKD------ 1IR3:A --HDFTV--KIGDFGMTRDIET-----DR---KGGKGLLPVRWMAPESLK-----DGVFT

1M14:A KT------PQHVKITDFGLAKLLG-----AEEKEYHAEGGKVPIKWMALESILH------ 1M14:A --TPQHV--KITDFGLAKLLGA-----EEKEYHAEGGKVPIKWMALESIL-----HRIYT

1GJO:A TE------NNVMKIADFGLARDIN-----NIDYYKKTTNGRLPVKWMAPEALFD------ 1GJO:A --ENNVM--KIADFGLARDINN-----IDYYKKTTNGRLPVKWMAPEALF-----DRVYT

Strand 8 Activation Loop Strand 8 Activation Loop

1CDK:A KG----YNKAVDWWALGVLIYEMAAG----------Y-PPFFAD-------QPIQIYEKI 1CDK:A NKAVDWWALGVLIYEMA-------------AGYPPF-----FADQPIQIYEKIVSG----

1O6L:A ND----YGRAVDWWGLGVVMYEMMCG----------R-LPFYNQ-------DHERLFELI 1O6L:A GRAVDWWGLGVVMYEMM-------------CGRLPF-----YNQDHERLFELILME----

1OMW:A VA----YDSSADWFSLGCMLFKLLRG----------H-SPFRQHKT--K--DKHEIDRMT 1OMW:A DSSADWFSLGCMLFKLL-------------RGHSPFR--QHKTKDKHEIDRMTLTM----

1H1W:A KS----ACKSSDLWALGCIIYQLVAG----------L-PPFRAG-------NEYLIFQKI 1H1W:A CKSSDLWALGCIIYQLV-------------AGLPPF-----RAGNEYLIFQKIIKL----

1MUO:A RM----HDEKVDLWSLGVLCYEFLVG----------K-PPFEAN-------TYQETYKRI 1MUO:A DEKVDLWSLGVLCYEFL-------------VGKPPF-----EANTYQETYKRISRV----

1TKI:A DV----VSTATDMWSLGTLVYVLLSG----------I-NPFLAE-------TNQQIIENI 1TKI:A STATDMWSLGTLVYVLL-------------SGINPF-----LAETNQQIIENIMNA----

1JKL:A EP----LGLEADMWSIGVITYILLSG----------A-SPFLGD-------TKQETLANV 1JKL:A GLEADMWSIGVITYILL-------------SGASPF-----LGDTKQETLANVSAV----

1A06 KP----YSKAVDCWSIGVIAYILLCG----------Y-PPFYDE-------NDAKLFEQI 1A06 SKAVDCWSIGVIAYILL-------------CGYPPF-----YDENDAKLFEQILKA----

1PHK PG----YGKEVDMWSTGVIMYTLLAG----------S-PPFWHR-------KQMLMLRMI 1PHK GKEVDMWSTGVIMYTLL-------------AGSPPF-----WHRKQMLMLRMIMSG----

1KWP:A EK----YDKSCDMWSLGVIMYILLCG----------Y-PPFYSNHG-----LAISPGMKT 1KWP:A DKSCDMWSLGVIMYILL-------------CGYPPFYS---NHGLAISPGMKTRIR----

1IA8:A EF----HAEPVDVWSCGIVLTAMLAG----------E-LPWDQPSD---------SCQEY 1IA8:A AEPVDVWSCGIVLTAML-------------AGELPW-----DQPSDSCQEYSDWKE----

1GNG:A TD----YTSSIDVWSAGCVLAELLLG----------Q-PIFPGD-------SGVDQLVEI 1GNG:A TSSIDVWSAGCVLAELL-------------LGQPIF-----PGDSGVDQLVEIIKVLGTP

1HCK KY----YSTAVDIWSLGCIFAEMVTR----------R-ALFPGD-------SEIDQLFRI 1HCK STAVDIWSLGCIFAEMV-------------TRRALF-----PGDSEIDQLFRIFRTLGTP

1JNK MG----YKENVDIWSVGCIMGEMVRH----------K-ILFPGR-------DYIDQWNKV 1JNK KENVDIWSVGCIMGEMV-------------RHKILF-----PGRDYIDQWNKVIEQLGTP

1HOW:A AP----WGCGADIWSTACLIFELITG----------D-FLFEPD--------DDDHIAQI 1HOW:A GCGADIWSTACLIFELI-------------TGDFLF------EPDDDDHIAQIIELLGEL

1LP4:A QD----YDYSLDMWSLGCMFAGMIFR----------KEPFFYGH-------DNHDQLVKI 1LP4:A DYSLDMWSLGCMFAGMI-------------FRKEPF----FYGHDNHDQLVKIAKVLGTD

1F3M:C KA----YGPKVDIWSLGIMAIEMIEG----------E-PPYLNE-------NPLRALYLI 1F3M:C GPKVDIWSLGIMAIEMI-------------EGEPPY-----LNENPLRALYLIATN----

1O6Y:A DS----VDARSDVYSLGCVLYEVLTG----------E-PPFTGD-------SPVSVAYQH 1O6Y:A DARSDVYSLGCVLYEVL-------------TGEPPF-----TGDSPVSVAYQH-VREDPI

1CSN RE----QSRRDDLEALGHVFMYFLRG----------S-LPWQGLKAATNKQKYERIGEKK 1CSN SRRDDLEALGHVFMYFL-------------RGSLPWQ--GLKAATNKQKYERIGEK----

1B6C:B NMKHFESFKRADIYAMGLVFWEIARRCSIGGIHEDYQ-LPYYDLVP--SDPSVEEMRKVV 1B6C:B FESFKRADIYAMGLVFWEIARRCSIGGIHEDYQLPYYDLVPSDPSVEEMRKVVCEQK---

2SRC GR----FTIKSDVWSFGILLTELTTK---------GR-VPYPGM-------VNREVLDQV 2SRC IKSDVWSFGILLTELTT-------------KGRVPY-----PGMVNREVLDQVERG----

1LUF:A NR----YTTESDVWAYGVVLWEIFSY---------GL-QPYYGM-------AHEEVIYYV 1LUF:A TESDVWAYGVVLWEIFS-------------YGLQPY-----YGMAHEEVIYYVRDG----

1IR3:A GV----FTTSSDMWSFGVVLWEITSL---------AE-QPYQGL-------SNEQVLKFV 1IR3:A TSSDMWSFGVVLWEITS-------------LAEQPY-----QGLSNEQVLKFVMDG----

1M14:A RI----YTHQSDVWSYGVTVWELMTF---------GS-KPYDGI-------PASEISSIL 1M14:A HQSDVWSYGVTVWELMT-------------FGSKPY-----DGIPASEISSILEKG----

1GJO:A RV----YTHQSDVWSFGVLMWEIFTL---------GG-SPYPGI-------PVEELFKLL 1GJO:A HQSDVWSFGVLMWEIFT-------------LGGSPY-----PGIPVEELFKLLKEG----

Helix F Helix F

1CDK:A VS-GK----------------------------------V--RF--P------SH----- 1CDK:A ----------------------------------KVRFPSH------------FSSDLKD

1O6L:A LM-EE----------------------------------I--RF--P------RT----- 1O6L:A ----------------------------------EIRFPRT------------LSPEAKS

1OMW:A LT-MA----------------------------------V--EL--P------DS----- 1OMW:A ----------------------------------AVELPDS------------FSPELRS

1H1W:A IK-LE----------------------------------Y--DF--P------EK----- 1H1W:A ----------------------------------EYDFPEK------------FFPKARD

1MUO:A SR-VE----------------------------------F--TF--P------DF----- 1MUO:A ----------------------------------EFTFPDF------------VTEGARD

1TKI:A MN-AE----------------------------------Y--TF--DE-E-AFKE----- 1TKI:A ----------------------------------EYTFDEEAFKE--------ISIEAMD

1JKL:A SA-VN----------------------------------Y--EF--ED-E-YFSN----- 1JKL:A ----------------------------------NYEFEDEYFSN--------TSALAKD

1A06 LK-AE----------------------------------Y--EF---D-SPYWDD----- 1A06 ----------------------------------EYEFDSPYWDD--------ISDSAKD

1PHK MS-GN----------------------------------Y--QF--GS-P-EWDD----- 1PHK ----------------------------------NYQFGSPEWDD--------YSDTVKD

1KWP:A RI-RM----------------------------------Y--EF--PNPE-WSEV----- 1KWP:A ---------------------------------MYEFPNPEWSEV--------SEEVKML

1IA8:A SD--W----------------------------------KEKKT--YL-N-PWKK----- 1IA8:A ---------------------KKTYLN----------PWKK------------IDSAPLA

1GNG:A IK-VLGT-PTRE-QIREMNP-N-YTEFK-----FPQIKAH--PWTKVF-R---PR----- 1GNG:A TREQIREM-------------NPNYTEFKFPQIKAHPWTKVFRPR--------TPPEAIA

1HCK FR-TLGTPD-EVVW-PG--VTSMP-DY-K-PSF-PKWARQ--DFSKVV-P----P----- 1HCK DEVVWPGV-------------TSMPDYKPSFPKWARQDFSKVVPP--------LDEDGRS

1JNK IE-QLGTPC-PE-FMKK--L-Q-PTVRNYVENR-PKYAGL--TFPKLF-P----DSLFPA 1JNK CPEFMKKLQPTVRNYVENRPKYAGLTFPKLFPDSLFPADSEHNKL--------KASQARD

1HOW:A IE-LLG-ELPSY-LL----R-N-GKYTRTFF---SKLKFW--PLEDVL-TEKYKF----- 1HOW:A PSYLLRNGKY----------------TRTFFSKLKFWPLEDVLTE-----KYKFSKDEAK

1LP4:A AK-VLGTDGLNV-YLNK--Y-R-IELDPQLEALVGRHSRK--PWLKFM-NADNQH----- 1LP4:A GLNVYL-------------NKYRIELDPQLEALVGRHSRKPWLKFMNADNQHLVSPEAID

1F3M:C AT-NG---------------------------------TP--EL--Q----NPEK----- 1F3M:C ----------------------------------GTPELQNPEK---------LSAIFRD

1O6Y:A VR-ED----------------------------------P--IP--PS-A-RHEG----- 1O6Y:A PPS------------------------------ARHEGLSA---------------DLDA

1CSN QS--T----------------------------------P--LR--E----LCAG----- 1CSN --------------------------------KQSTPLRELCAG---------FPEEFYK

1B6C:B CEQKL----------------------------------R--PN--IP-N-RWQS----- 1B6C:B ----------------------------------LRPNIPNRWQS-------CEALRVMA

2SRC ER-GY---------------------------------RM--PC--P------PE----- 2SRC ----------------------------------YRMPCPPE-----------CPESLHD

1LUF:A RD-GN---------------------------------IL--AC--P------EN----- 1LUF:A ----------------------------------NILACPEN-----------CPLELYN

1IR3:A MD-GG---------------------------------YL--DQ--P------DN----- 1IR3:A ----------------------------------GYLDQPDN-----------CPERVTD

1M14:A EK-GE---------------------------------RL--PQ--P------PI----- 1M14:A ----------------------------------ERLPQPPI-----------CTIDVYM

1GJO:A KE-GH---------------------------------RM--DK--P------AN----- 1GJO:A ----------------------------------HRMDKPAN-----------CTNELYM

1CDK:A -------FSSDLKDLLRNLLQVDLTKRFGN-LKDGVNDIKNHKWFAT 1CDK:A LLRN----LLQVDLTKRFGNLKD-GVNDIKNHKWFAT

1O6L:A -------LSPEAKSLLAGLLKKDPKQRLGG-GPSDAKEVMEHRFFLS 1O6L:A LLAG----LLKKDPKQRLGGGPS-DAKEVMEHRFFLS

1OMW:A -------FSPELRSLLEGLLQRDVNRRLGC-LGRGAQEVKESPFFRS 1OMW:A LLEG----LLQRDVNRRLGC-LGRGAQEVKESPFFRS

1H1W:A -------FFPKARDLVEKLLVLDATKRLGCEEMEGYGPLKAHPFFES 1H1W:A LVEK----LLVLDATKRLGCEEMEGYGPLKAHPFFES

1MUO:A -------VTEGARDLISRLLKHNPSQRPM------LREVLEHPWITA 1MUO:A LISR----LLKHNPSQRP------MLREVLEHPWITA

1TKI:A -------ISIEAMDFVDRLLVKERKSRMT------ASEALQHPWLKQ 1TKI:A FVDR----LLVKERKSRM------TASEALQHPWLKQ

1JKL:A -------TSALAKDFIRRLLVKDPKKRMT------IQDSLQHPWIKP 1JKL:A FIRR----LLVKDPKKRM------TIQDSLQHPWIKP

1A06 -------ISDSAKDFIRHLMEKDPEKRFT------CEQALQHPWIAG 1A06 FIRH----LMEKDPEKRF------TCEQALQHPWIAG

1PHK -------YSDTVKDLVSRFLVVQPQKRYT------AEEALAHPFFQQ 1PHK LVSR----FLVVQPQKRY------TAEEALAHPFFQQ

1KWP:A -------SEEVKMLIRNLLKTEP-TQRMT------ITEFMNHPWIMQ 1KWP:A IRNL----LKTEPT-QRM------TITEFMNHPWIMQ

1IA8:A -------IDSAPLALLHKILVENPSARIT------IPDIKKDRWYNK 1IA8:A LLHK----ILVENPSARI------TIPDIKKDRWYNK

1GNG:A -------TPPEAIALCSRLLEYTPTARLT------PLEACAHSFFDE 1GNG:A LCSR----LLEYTPTARL------TPLEACAHSFFDE

1HCK -------LDEDGRSLLSQMLHYDPNKRIS------AKAALAHPFFQD 1HCK LLSQ----MLHYDPNKRI------SAKAALAHPFFQD

1JNK DSEHNKLKASQARDLLSKMLVIDPAKRIS------VDDALQHPYINV 1JNK LLSK----MLVIDPAKRI------SVDDALQHPYINV

1HOW:A ----SKDEAKEISDFLSPMLQLDPRKRAD------AGGLVNHPWLKD 1HOW:A EISDFLSPMLQLDPRKRA------DAGGLVNHPWLKD

1LP4:A ------LVSPEAIDFLDKLLRYDHQERLT------ALEAMTHPYFQQ 1LP4:A FLDK----LLRYDHQERL------TALEAMTHPYFQQ

1F3M:C -------LSAIFRDFLNRCLDMDVEKRGS------AKELLQHQFLKI 1F3M:C FLNR----CLDMDVEKRG------SAKELLQHQFLKI

1O6Y:A -------LSADLDAVVLKALAKNPENRYQT-----AAEMRAD-LVRV 1O6Y:A VVLK----ALAKNPENRY------QTAAEMRADLVRV

1CSN -------FPEEFYKYMHYARNLAFDATPD------YDYLQGL-FSKV 1CSN YMHY---ARNLAFDATPD-------YDYLQGLFSKV-

1B6C:B -----CEALRVMAKIMRECWYANGAARLT------ALRIKKT-LSQL 1B6C:B KIMRE---CWYANGAARL------TALRIKKTLSQL-

2SRC -------CPESLHDLMCQCWRKEPEERPT------FEYLQAF-LEDY 2SRC LMCQ----CWRKEPEERP------TFEYLQAFLEDY-

1LUF:A -------CPLELYNLMRLCWSKLPADRPS------FCSIHRI-LQRM 1LUF:A LMRL----CWSKLPADRP------SFCSIHRILQRM-

1IR3:A -------CPERVTDLMRMCWQFNPKMRPT------FLEIVNL-LKDD 1IR3:A LMRM----CWQFNPKMRP------TFLEIVNLLKDD-

1M14:A -------CTIDVYMIMVKCWMIDADSRPK------FRELIIE-FSKM 1M14:A IMVK----CWMIDADSRP------KFRELIIEFSKM-

1GJO:A -------CTNELYMMMRDCWHAVPSQRPT------FKQLVED-LDRI 1GJO:A MMRD----CWHAVPSQRP------TFKQLVEDLDRI-

Helix I Helix I
